# Retrospective Analysis of Surgical Outcomes on Axial Length Elongation in Eyes with Posterior and Combined Persistent Fetal Vasculature

**DOI:** 10.3390/ijms24065836

**Published:** 2023-03-19

**Authors:** Heng-Chiao Huang, Chien-Hsiung Lai, Eugene Yu-Chuan Kang, Kuan-Jen Chen, Nan-Kai Wang, Laura Liu, Yih-Shiou Hwang, Chi-Chun Lai, Wei-Chi Wu

**Affiliations:** 1 Department of Ophthalmology, Chang Gung Memorial Hospital, Chiayi 613, Taiwan; 2 College of Medicine, Chang Gung University, Taoyuan 333, Taiwan; 3 Department of Ophthalmology, Chang Gung Memorial Hospital, Taoyuan 333, Taiwan; 4 Department of Ophthalmology, Edward S. Harkness Eye Institute, Columbia University Medical Center, New York, NY 10032, USA; 5 Department of Ophthalmology, Chang Gung Memorial Hospital, Keelung 204, Taiwan

**Keywords:** axial length, outcomes, persistent fetal vascular vasculature, persistent hyperplastic primary vitreous, risk factors, vitrectomy

## Abstract

This study aims to investigate the outcomes and risk factors associated with poor vision (vision less than counting fingers, 2.0 logMAR, Snellen vision 20/2000) in patients with posterior or combined persistent fetal vasculature (PFV), with or without surgery. We retrospectively reviewed the medical records of patients who were diagnosed with PFV from January 2008 to April 2021. We included 51 eyes of 44 patients who presented with PFV, of which 38 eyes underwent surgical correction (pars plicata/plana vitrectomy, with or without lensectomy, and intraocular lens implantation) at the median age of 6.0 months (range: 0.7 to 82.0). The mean follow-up was 68.8 months ± 38.0 months. The axial length change in the eyes undergoing surgery was significantly higher than the eyes without surgery (*p* = 0.025). Initial anterior chamber collapse and retinal detachment were associated with poor vision (*p* = 0.006 and *p* = 0.002, respectively). In addition, 37% of eyes with posterior or combined PFV had vision better than counting fingers. Surgery for eyes with PFV could result in better eye growth. Visual outcomes remained poor and were associated with the level of macular abnormality. Initial anterior chamber collapse and retinal detachment at presentation were the risk factors for poor visual outcomes. Vitrectomy for selected PFV eyes is valuable and associated with a better cosmetic outcome (better eye growth).

## 1. Introduction

Persistent fetal vasculature (PFV), first described by Reese [1] in 1955, is a congenital ocular syndrome caused by the failure of normal regression of the intraocular blood vessels of a foetus. The disease can present as retrolental tissue connecting anterior to posterior segment, lens opacification, elongation of ciliary process, retinal detachment, macular hypoplasia and dysplasia, optic nerve hypoplasia or dysplasia, and malformation in the size of the globe [2]. The PFV patients are typically unilateral, and about 10% of cases have bilateral involvement [2]. PFV is categorised into anterior, posterior, and combined types according to the location of the remaining vasculature [2,3]. Anterior PFV presents as a shallowing of the anterior chamber (AC), a congenital cataract, and a fibrovascular membrane surrounding the lens capsule that may cause traction force over the ciliary process. The hyaloidal stalk that connects the posterior lens surface to the optic nerve head is the main clinical feature of posterior PFV. The presence of the stalk causes vitreoretinal traction over the posterior pole, which can lead to tractional retinal detachment. Combined PFV is the most common type and is characterised by the presence of both anterior and posterior PFV [4,5,6]. Microphthalmia is associated with poor visual outcome [3,4,7].

Untreated severe PFV can result in corneal opacity, shallow AC, secondary angle-closure glaucoma, spontaneous intraocular haemorrhage, retinal detachment, and phthisis bulbi. The visual prognosis is poor, and a patient may even require enucleation, due to pain and uncontrolled intraocular pressure (IOP) [2,4]. The method of surgical intervention is determined by the involvement of the anterior or posterior segment in PFV. The early treatment of PFV is necessary to eliminate media opacities and traction force to prevent glaucoma or phthisis bulbi, improve cosmetic outcomes, and, if possible, restore functional vision [8,9].

The severity and clinical presentations of PFV vary widely. The value of surgical interventions and clinical indicators used as predictors of PFV outcomes remain uncertain. No consensus has been reached regarding the optimal surgical management of eyes with PFV. Although previous studies have demonstrated outcomes in eyes with PFV, relatively few studies have determined the prognostic factors for visual outcomes, and the axial length (AL) elongation [7,9,10,11,12,13,14]. This study thus will assess the anatomical and functional outcomes in eyes with PFV, with or without surgery, to determine the value of surgical interventions, and the initial characteristics that can be used to predict poor visual outcomes.

## 2. Results

**Figure 1:**
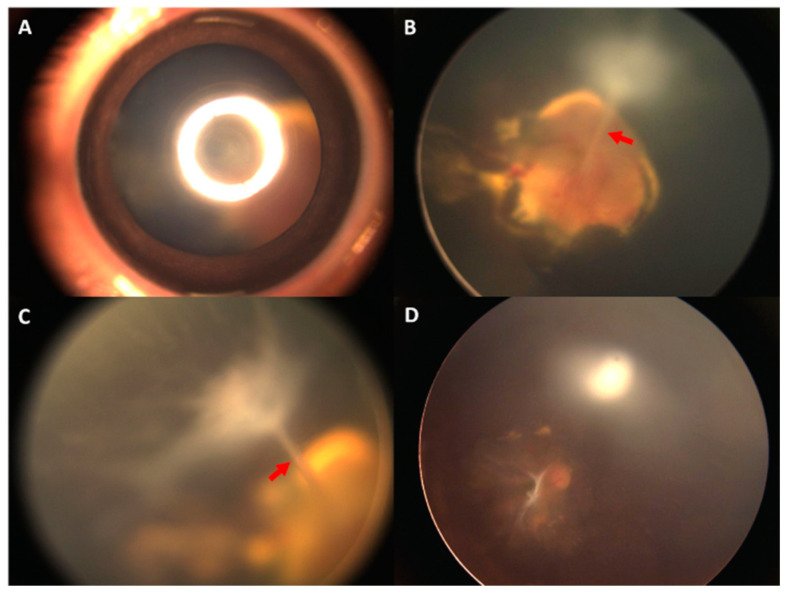
Morphologic changes after vitrectomy in a patient with combined persistent fetal vasculature. (**A**–**C**) Photograph of the external eye and fundus of a 3-month-old boy with unilateral combined persistent fetal vasculature before operation. Focal posterior lens opacity was observed. Retrolental stalk tissue (red arrow), extending from the optic disc to the back of lens, and a centrally located vascular retina were noted. (**D**) A 23G vitrectomy was performed to remove the stalk tissue. The remnant on the optic disc is seen in the photograph of the fundus.

We included 51 eyes of 44 patients with PFV, 38 of which underwent surgical correction for PFV. The patient demographic data are shown in Table 1 and Supplementary Table S1. The median age at diagnosis was 5.0 months (range: 0.7 to 81.0), and the median age at operation was 6.0 months (range: 0.7 to 82.0). Twenty-six (59.1%) patients were male, and seven patients (15.9%) had bilateral PFV. In surgical eyes, 2 eyes (5.3%) had posterior PFV, and 36 eyes (94.7%) had combined PFV, whereas in nonsurgical eyes, 9 (69.2%) eyes had posterior PFV, and 4 eyes (30.8%) were combined type. Preoperatively, the initial characteristics of AC collapse, cataract, and retinal detachment were significantly different between surgical and nonsurgical eyes. The mean AL was significantly shorter in surgical eyes (18.3 ± 3.0 mm) than in nonsurgical eyes (20.9 ± 1.4 mm; *p* = 0.009). The horizontal corneal diameter was significantly smaller in surgical eyes (*p* = 0.046), but the vertical diameter was not (*p* = 0.172). The eyes that required a surgical correction tended to be the combined type with one of the characteristics of AC collapse, cataract, retinal detachment, or smaller eyes. Of the 38 eyes that underwent surgical correction, 12 eyes (31.6%) received pars plicata vitrectomy (PPV), 3 eyes (7.9%) received pars plicata lensectomy (PPL), and 17 eyes (44.7%) had PPV combined with PPL. Only two eyes (5.3%) received PPV, PPL, and an intraocular lens (IOL) implantation during the primary surgery. An example of fundus morphologic changes after PPV for stalk removal in a 3-month-old boy with unilateral combined PFV is shown in Figure 1. The change in AL of the lesion eye was 2.97 mm (increased from 16.5 to 19.47 mm) in the follow-up period of 51 months.

Preoperatively, the ocular parameters of lesions and normal eyes are shown in Table 2. The mean AL was significantly shorter in the lesion eyes (19.1 ± 2.9 mm) than in the normal eyes (20.4 ± 1.7 mm; *p* = 0.036). In terms of corneal size, both horizontal (10.4 ± 1.1 vs. 11.6 ± 0.7 mm) and vertical corneal diameters (10.2 ± 0.8 vs. 11.3 ± 0.8 mm) were significantly smaller in the lesion eyes (*p* < 0.001). No significant difference in the IOP of the groups was found (11.0 ± 4.8 vs. 12.0 ± 3.9 mmHg; *p* = 0.167). The comparison showed that the PFV eyes were characterised by microphthalmia, which may cause cosmetic problems for the patients.

**Figure 2:**
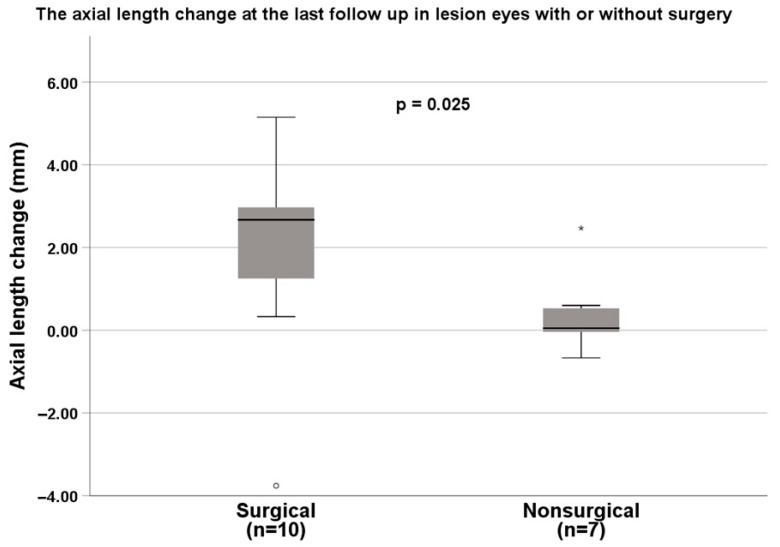
Axial length change at the last follow-up in the lesion eyes with or without surgery. The eyes with PFV that received a surgical correction had significantly more axial length elongation. Mann–Whitney *U* test. * Extreme outliers.

The mean follow-up period was 68.8 months ± 38.0 months (range: 9.0 to 163.0). At the final follow-up, the best-corrected visual acuity (BCVA) was evaluated in 39 (76.5%) of the 51 eyes. In the surgical group, 10 eyes (26.3%) had favourable vision, which was indicated by a BCVA better than, or equal to, counting fingers (2.0 logMAR, Snellen vision of 20/2000; Table 3). Seventeen eyes (44.7%) had poor vision, 4 of them were light perception, and 11 of them were no light perception (NLP). In the nonsurgical group, nine eyes (69.2%) had favourable vision, and three eyes (23.1%) had poor vision, with two eyes with light perception and none with NLP. The surgical eyes had significantly poorer vision than the nonsurgical group (*p* = 0.041). The possible cause was that the surgical group may have had more significant structure involvement, such as AC collapse or retinal detachment, as previously mentioned, and thus required surgical correction. At the final follow-up, only one eye (2.6%) developed glaucoma, and five eyes (13.2%) had phthisis bulbi in the surgical group, and none of the above was observed in the nonsurgical eyes. The final lens status and retinal detachment at the final visit were significantly different between the two groups (*p* < 0.001 and *p* = 0.007, respectively). No eyes developed endophthalmitis or required enucleation. Although surgical intervention was intended to correct structure anomalies and prevent complications, there were still higher rates of poor outcomes in the surgical eyes. The mean of the final AL did not significantly differ between the surgical (20.3 ± 3.6 mm) and nonsurgical eyes (22.0 ± 1.0 mm; *p* = 0.446). However, the lesion eyes receiving surgery had a significantly higher increase in AL than the eyes without surgery (2.0 ± 2.4 and 0.4 ± 1.0, respectively; *p* = 0.025, (Figure 2)). This result means that the eyes after surgical intervention had gained better eye growth and, therefore, a better cosmetic outcome (less microphthalmia).

**Figure 3:**
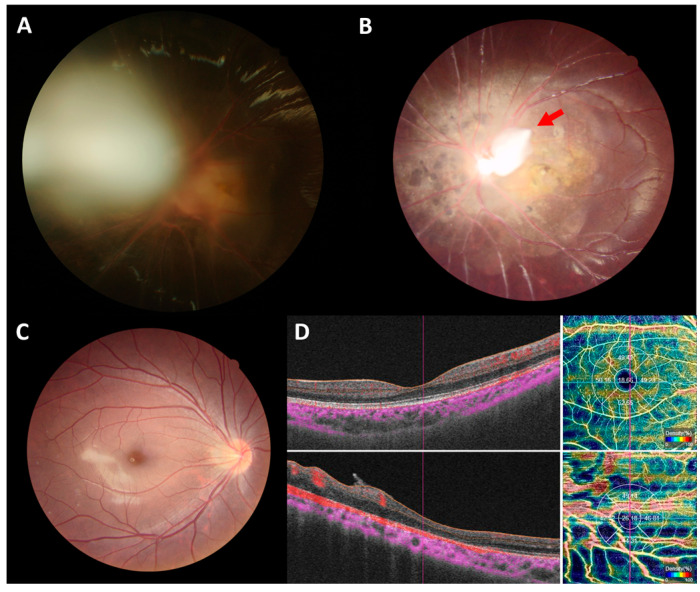
Changes in fundus after vitrectomy in a patient with combined persistent fetal vasculature. (**A**) Photograph of a 7-year-old girl’s eye with unilateral combined persistent fetal vasculature, with focal lens opacity, and a stalk extending from the optic nerve to the posterior lens capsule before the operation. (**B**) Stalk remnant (red arrow) is seen around the disc, with a para-disc dysplastic tissue in the left eye, after vitrectomy for stalk removal. Her final BCVA was 1.52 logMAR (Snellen ratio of 0.03). (**C**) Photograph of the fundus of the normal eye. (**D**) OCTA showed diffuse decreased vessel density, and a thin retina with foveal hypoplasia (lower panel), distinguishing it from the normal eye (upper panel).

Figure 3 shows the fundus images of a seven-year-old girl with unilateral combined PFV after PPV. An optical coherence tomography angiography (OCTA) revealed decreased vessel density, and a thin retina with foveal hypoplasia. Her visual acuity (VA) was 1.52 logMAR (Snellen equivalent of 0.03) after surgery. Her vision was associated with the level of macular abnormality.

As shown in Table 4, the initial clinical characteristics of PFV were associated with poor vision outcomes (worse than counting fingers) in our study patients. Initial AC collapse and retinal detachment were risk factors for poor vision (*p* = 0.006 and *p* = 0.002, respectively). Corneal opacity and cataracts were not significantly associated with poor outcomes. The surgical eyes tended to have the initial characteristics of AC collapse and retinal detachment, which were associated with poor vision, therefore the surgical eyes had poorer outcomes than the nonsurgical eyes.

The median of AL was 17.5 mm and 21.2 mm in the eyes with poor and favourable vision, respectively (Supplementary Table S2). The AL was significantly shorter in the eyes with poor vision (*p* = 0.001). In terms of corneal size, the median horizontal and vertical corneal diameters did not significantly differ between the two groups (*p* = 0.126 and *p* = 0.245, respectively).

## 3. Discussion

PFV is a significantly blinding disease in paediatric patients as a result of the anatomic malformation and secondary complications of glaucoma, retinal detachment, and phthisis. This study observed significantly shorter AL and smaller corneal diameter in the lesion eyes. The eyes receiving surgery had significantly higher increases in AL than the eyes without surgery (2.0 ± 2.4 vs. 0.4 ± 1.0 mm; *p* = 0.025). Only seven eyes (13.7%) had a final VA > 20/400. The initial characteristics of AC collapse and/or retinal detachment were associated with significantly poor visual outcomes in eyes with PFV, whereas corneal opacity and cataracts were not significant factors for poor visual outcomes. In addition, AL was significantly shorter in the eyes with poor vision, which is consistent with the findings of previous studies that microphthalmia is a risk factor for poor prognosis [7,9,15]. Our study suggests that surgery in eyes with PFV may result in an acceptable anatomical outcome, and improved eye growth; however, the visual outcomes remained poor because of the presence of frequent macular involvement in the eyes with PFV.

Indications for surgical interventions in patients with PFV vary depending on the patients’ clinical characteristics. Visual axis obscuration, such as secondary cataract, retrolental fibrous membrane, and intraocular haemorrhage, is indicated. Other indications include a stalk with vitreoretinal traction, retinal detachment, progressive AC shallowing, and uncontrolled glaucoma [2]. Surgery may not be required in the eyes with a mildly affected stalk, without media opacities, or with advanced abnormalities because of the limited potential for vision improvement [7]. In our study, corneal opacity and/or AC shallowing were indicators for surgery in the eyes with PFV. Although the potential for visual improvement is limited due to the frequency of retinal detachment pushing the iris forward, surgery can help prevent secondary glaucoma, or phthisis bulbi, and subsequently improve cosmetic outcomes. The prognosis after surgical intervention varies depending on the type and extent of PFV. Postoperative visual outcomes are more favourable in eyes with stalk and minimal macular involvement.

The majority of PFV cases are sporadic and unilateral, and less than 10% of patients with PFV have bilateral involvement [2,8,16]. In this study, seven (15.9%) patients had bilateral PFV, which is slightly higher than the reported data. In previous studies, the most common type of PFV was combined (78% in this study), which is typically associated with a poor prognosis, even with appropriate management [10,11,16]. The clinical heterogeneity among patients with PFV explains the disparities in visual outcomes across studies (Supplementary Table S3) [9,11,12,13,14]. Anteby et al. [17] found that with or without surgery, 24% (21 of 87) of patients with PFV can achieve a VA of 6/60 or better. Hunt et al. [10] reported that 6 (18%) of the 33 eyes that received surgery achieved a VA of 6/60 or better. Khandwala et al. [14] found that 3 (21%) of the 14 eyes with mild combined PFV that received lensectomy, with or without IOL implantation, had a VA of 20/200 or better. In our study, only 1 (3%) of the 38 eyes that received surgery achieved a VA of 20/200 or better. The visual outcomes were relatively poor, possibly because all the patients had posterior-involved PFV with macular involvement.

Another factor that may be associated with the visual prognosis is the time of surgical intervention. Due to the development of surgical treatment methods, early surgical intervention is recommended if an operation is indicated [3,7,10]. Significant reversal of retinal dragging and a reattached retina was observed in children with surgical interventions before the age of 13 months [18]. Hunt et al. [10] suggested that a surgery performed before 77 days of age was 13 times more likely to achieve a VA of finger counting or better. Retinal folding, vitreomacular traction, and retinal detachment may manifest fibrovascular changes and a loss of plasticity if left untreated for a certain period. In this study, the median age at operation was 6.0 months (range: 0.7 to 82.0). In total, 26 (68%) of the 38 eyes received surgery before 13 months. Of the 17 eyes with a measurable VA, 6 (35%) had a VA of 2.0 logMAR or better. By contrast, the 12 eyes (32%) that received surgery after 13 months of age, and 4 (40%) of the 10 eyes with a measurable VA, had a VA of 2.0 logMAR or better. These results are inconsistent with those of previous studies. This may be because eyes with more severe and advanced pathology are clinically detected earlier and thus treated sooner.

The factors associated with visual outcomes include age-at-presentation, age-at-surgical intervention, bilaterality, type of PFV, and secondary complications. Zhang and colleagues [19] reported that the AL in the eyes with unilateral cataracts and PFV is significantly shorter in patients younger than 24 months, compared with patients with fellow eyes. Moreover, an AL shorter than 15mm and a 3.5 mm AL difference between the two eyes are associated with poorer visual outcomes [11]. In this study, we found that the eyes with PFV had a shorter AL, which is associated with poor vision. These findings are consistent with those of previous studies. The lesion eyes had significantly increased AL after surgical intervention, compared with the lesion eyes without surgical intervention. This finding indicates that the eyes continue to grow after appropriately releasing the traction through the surgery, thereby achieving better cosmetic outcomes. In addition, the initial presentations of AC collapse and/or retinal detachment are key factors associated with poor visual outcomes. The collapse of the AC can be caused by the swelling of the lens, or by contracture of the retrolental tissue with anterior shifting of the lens–iris diaphragm [2]. Retinal detachment is caused by the traction force of the persistent intraocular vasculature connecting the optic nerve and the back of the lens surface. Surgically reattaching the retina in patients with congenital retinal nonattachment is challenging. Because the stalk tissue can limit eye growth, short AL is observed in such cases.

As strengths, this retrospective study enrolled a relatively large number of patients with a rare disease, and included a long follow-up period; however, this study has several limitations. First, the study design was retrospective, where a single surgeon performed all the procedures. Second, the evaluation of visual outcomes may have been limited because a VA was not measurable in several children. Third, the AL was not measurable in the eyes with severe anatomic abnormalities. Finally, a selection bias may have existed because a surgery might not be performed in the eyes with advanced PFV, such as PFV involving severe closed-funnel retinal detachment, or optic nerve hypoplasia. This could only be addressed in the future prospective randomised trials. Because PFV is a rare disease, a large multicentre collaborative study on the optimal management of PFV is warranted.

## 4. Materials and Methods

### 4.1. Study Population 

**Figure 4:**
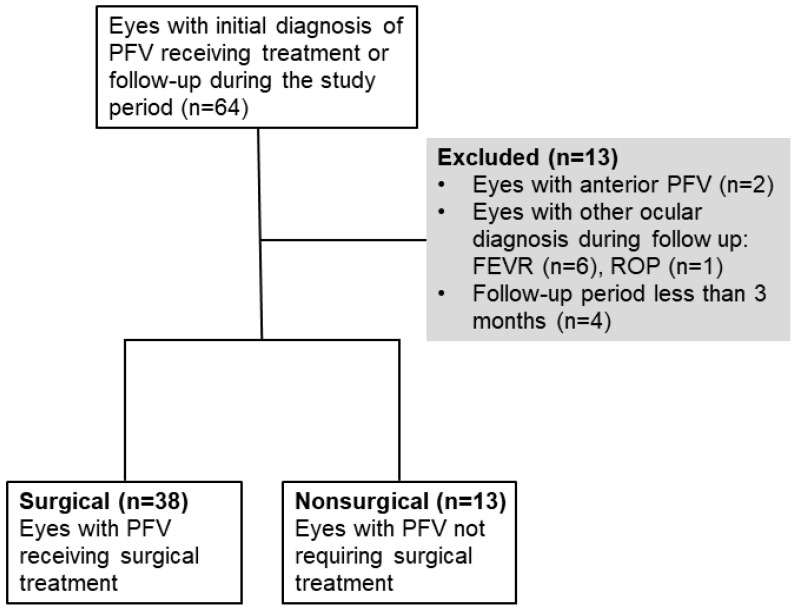
Flowchart showing the inclusion and exclusion of the patients’ eyes during the study period.

This study was approved (IRB 202201840B0) by the Institutional Review Board of Chang Gung Memorial Hospital, in Taoyuan, Taiwan, and was conducted in accordance with the Declaration of Helsinki. This was a retrospective chart review case study. Patients who were diagnosed with either unilateral or bilateral PFV from January 2008 to April 2021 were included in this study. PFV was defined as a failure of normal regression of the fetal intraocular vasculature, which was detected using slit lamp examination, or indirect ophthalmoscopy. Patients with posterior or combined PFV, with or without surgical intervention, were included. Patients with a follow-up period of less than 3 months, and with other diagnoses, such as retinoblastoma, Norrie disease, familial exudative vitreoretinopathy, and retinopathy of prematurity, were excluded (Figure 4). Patient demographic data, specifically age, gender, family and medical history, laterality, age at operation, AL, corneal size, IOP, surgical methods, postoperative complications, and the VA at the final visit, were recorded.

### 4.2. Ocular Examination and Grouping

Under general anaesthesia, all patients received a detailed ophthalmologic examinations, including an IOP measurement with applanation tonometry, slit lamp biomicroscopy, dilated fundoscopic exam, B-scan ultrasound, colour fundus photography, and fluorescein angiography (RetCam 3; Natus Medical, Inc., Pleasanton, CA, USA). Due to the postoperative low vision, the BCVA was replaced with the Snellen ratio, in accordance with the methods proposed by Bach et al. [20]; these ratios were further converted to the logarithm of the minimum angle of resolution (logMAR) values. The patients with poor vision, limited to light perception or worse, were not converted. For the purpose of analysis, poor vision was defined as a BCVA worse than 2.0 logMAR (i.e., finger counting vision), and favourable vision was indicated by a BCVA equal to, or better than, 2.0 logMAR [10]. The lesion eyes were defined as unilateral PFV and both eyes having bilateral PFV, whereas normal eyes were defined as normal fellow eyes of the unilateral PFV. The lesion eyes were divided into two groups, surgical and nonsurgical for analysis.

### 4.3. Surgical Procedures

All surgeries were planned and performed by a single surgeon (W.-C.W.) at a tertiary medical centre. Standardised three-port vitrectomies using pars plicata/plana (23-gauge or 25-gauge), with or without lensectomy, were conducted in eyes with PFV. An IOL implantation was considered an optional procedure that could be performed in a subsequent surgery. Indications for surgical interventions included a visually threatened cataract or retrolental membrane, progressive AC shallowing, a stalk that had significant vitreoretinal traction, retinal detachment, and vitreous haemorrhage. The fibrovascular stalk was divided using a high-speed cutter, and the posterior hyaloid membrane was removed to release the vitreoretinal traction force. In addition, a vitrectomy was performed around the residual stalk to release the posterior traction. A diathermy was used for bleeding control, if necessary. Air–fluid exchange was performed according to the patients’ condition before the end of the operation.

### 4.4. Statistical Methods

Descriptive statistics (i.e., means, standard deviations, and percentages) were computed for demographic and clinical variables. For the comparison of surgical and nonsurgical PFV, a Fisher’s exact test was used in qualitative data and a Mann–Whitney *U* test was used to compare continuous variables with non-normal distributions. An independent *t* test was used to compare initial ocular measurements in lesion eyes and normal eyes. To explore the risk factors for poor vision, we conducted a multivariable logistic analysis by a backward selection of the recorded initial characteristics. The odds ratios (ORs) with 95% confidence intervals (CI) were adjusted for sex and age at diagnosis. A *p* value of <0.05 was considered statistically significant.

## 5. Conclusions

This study found that 37% of the eyes with posterior or combined PFV achieved vision better than counting fingers. An initial shallow AC and retinal detachment were found to be risk factors for poor visual outcomes. Surgery is valuable because it can facilitate the eye growth in selected PFV, which can then yield improved cosmetic outcomes.

## Figures and Tables

**Table 1:** Patient Demographic and Clinical Characteristics and Types of Persistent Fetal Vasculature of the Eye.

Characteristic	Surgical (*n* = 38)	Nonsurgical (*n* = 13)	*p* Value
Types of PFV, *n* (%)			<0.001 *
Posterior	2 (5.3)	9 (69.2)	
Combined	36 (94.7)	4 (30.8)	
Initial status, *n* (%)			
Cornea opacity	11 (28.9)	1 (7.7)	0.151 *
AC collapse	19 (50.0)	1 (7.7)	0.008 *
Cataract	36 (94.7)	4 (30.8)	<0.001*
Retinal detachment	21 (55.3)	1 (7.7)	0.003 *
Stalk	37 (97.4)	12 (92.3)	0.449 *
Macular dragging	36 (94.7)	11 (84.6)	0.266 *
Axial length, mm	18.3 ± 3.0	20.9 ± 1.4	0.009 ^†^
Missing or unmeasurable	15	2	
Horizontal corneal diameter, mm	10.2 ± 1.1	11.0 ± 0.8	0.046 ^†^
Missing or unmeasurable	2	1	
Vertical corneal diameter, mm	10.1 ± 0.8	10.6 ± 0.5	0.172 ^†^
Missing or unmeasurable	2	4	

AC: anterior chamber; PFV: persistent fetal vasculature. * Fisher’s exact test. ^†^ Mann–Whitney *U* test.

**Table 2:** Ocular Parameters in Lesion and Normal Eyes.

	Lesion Eyes (*n* = 51)	Normal Eyes (*n* = 37)	*p* Value *
Axial length, mm	19.1 ± 2.9	20.4 ± 1.7	0.036
Missing or unmeasurable	17	7	
Horizontal corneal diameter, mm	10.4 ± 1.1	11.6 ± 0.7	<0.001
Missing or unmeasurable	3	3	
Vertical corneal diameter, mm	10.2 ± 0.8	11.3 ± 0.8	<0.001
Missing or unmeasurable	6	6	
IOP, mmHg	11.0 ± 4.8	12.0 ± 3.9	0.167
Missing or unmeasurable	6	7	

IOP: intraocular pressure. * Independent *t* test.

**Table 3:** Final Outcomes and Status for Surgical and Nonsurgical Eyes with Persistent Fetal Vasculature.

Characteristics	Surgical (*n* = 38)	Nonsurgical (*n* = 13)	*p* Value
Final visual acuity of lesion eyes			0.041 *
Favourable vision (CF and better than CF)	10 (26.3)	9 (69.2)	
Poor vision (worse than CF)	17 (44.7)	3 (23.1)	
Missing or unmeasurable	11 (28.9)	1 (7.7)	
Final status, *n* (%)			
Glaucoma	1 (2.6)	0 (0.0)	1.00 *
Phthisis bulbi	5 (13.2)	0 (0.0)	0.311 *
Corneal opacity	14 (36.8)	4 (30.8)	0.750 *
Lens			<0.001 *
phakia	7 (18.4)	13 (100.0)	
Aphakia	20 (52.6)	0 (0.0)	
Pseudophakia	11 (28.9)	0 (0.0)	
Fundus			0.007 *
No retinal detachment	16 (42.1)	12 (92.3)	
Retinal detachment	16 (42.1)	1 (7.7)	
No view	6 (15.8)	0 (0.0)	
Final axial length, mm	20.3 ± 3.6	22.0 ± 1.0	0.446 ^†^
Missing or unmeasurable	23	4	
Axial length increase, mm	2.0 ± 2.4	0.4 ± 1.0	0.025 ^†^
Missing or unmeasurable	28	6	

CF: counting fingers. * Fisher’s exact test. ^†^ Mann–Whitney *U* test.

**Table 4:** Multivariable Analysis Results for Factors Associated with Poor Visual Outcomes in Patients with Persistent Fetal Vasculature of the Eye.

Factors	Poor Vision (Worse than CF) (*n* = 20)	Unadjusted OR (95% CI)	*p* Value	Adjusted OR * (95% CI)	*p* Value
Corneal opacity, n (%)			0.299		0.493
Yes	6 (30.0)	2.286 (0.480–10.883)		1.758 (0.350–8.819)	
No	14 (70.0)	1.000		1.000	
AC collapse, n (%)			0.004		0.006
Yes	12 (60.0)	12.750 (2.291–70.967)		34.256 (2.750–426.766)	
No	8 (40.0)	1.000		1.000	
Cataract, n (%)			0.128		0.335
Yes	17 (85.0)	3.306 (0.708–15.438)		2.334(0.416–13.087)	
No	3 (15.0)	1.000		1.000	
Retinal detachment, n (%)			0.002		0.002
Yes	14 (70.0)	12.444 (2.614–59.251)		26.409 (3.473–200.837)	
No	6 (30.0)	1.000		1.000	

AC: anterior chamber; CF: counting fingers. * Odds ratios (ORs) with 95% confidence intervals (CI) after adjusted for sex and age at diagnosis using a logistic regression analysis.

## Data Availability

The data presented in this study are available on request from the corresponding author. The data are not publicly available due to privacy and ethical restrictions.

## Supplementary Materials

The following supporting information can be downloaded at: https://www.mdpi.com/article/10.3390/ijms24065836/s1.

## Abbreviations

AC	Anterior chamber
AL	Axial length
BCVA	Best-corrected visual acuity
IOL	Intraocular lens
IOP	Intraocular pressure
PFV	Persistent fetal vasculature
PPL	Plicata lensectomy
PPV	Plicata vitrectomy
