# Supplementary material for: Retrospective Analysis of Surgical Outcomes on Axial Length Elongation in Eyes with Posterior and Combined Persistent Fetal Vasculature
Source: Int J Mol Sci. 2023 Mar 19;24(6):5836. doi: 10.3390/ijms24065836 (PMC10057662; doi:10.3390/ijms24065836)
Supplement: Supplementary file 1 [file ijms-24-05836-s001.zip › ijms-2200520-supplementary.pdf]

Supplementary Tables

Table S1. Patient Demographic and Surgical Methods of Persistent Fetal Vasculature of the Eye.

Characteristic	Value
Patients (Eyes)	44 (51)
Age at diagnosis, month (median)	5.0 (range: 0.7-81.0)
Age at operation, month (median)	6.0 (range: 0.7-82.0)
Male, n (%)	26 (59.1)
Laterality, Bilateral, n (%)	7 (15.9)
Lesion eyes, n (%)	51
Right	27 (52.9)
Left	24 (47.1)
Surgical methods, n (%)	n=38
PPV	12 (31.6)
PPL	3 (7.9)
PPV + PPL	17 (44.7)
PPV + PPL + IOL implant	2 (5.3)
CE-IOL	4 (10.5)

IOL: intraocular lens; CE-IOL: cataract extraction and IOL implantation; PPL: pars plicata lensectomy; PPV: pars plicata vitrectomy

Table S2. Ocular Parameters Associated with Poor Visual Outcomes in Patients with Persistent Fetal Vasculature of the Eye.

Factors	Poor Vision (worse than CF) (n=20)	Favorable Vision (CF and better than CF) (n=19)	*p* value*
Initial axial length, mm			
Mean	16.6±3.0	21.0±1.8	
Median (Q1, Q3)	17.5(13.8, 19.3)	21.2 (19.2, 22.5)	0.001
Missing or unmeasurable	13	2	
Horizontal corneal diameter, mm			
Mean	10.5±1.0	10.8±0.8	
Median (Q1, Q3)	10.5 (9.9, 11.0)	11.0 (10.4, 11.4)	0.126
Missing or unmeasurable	2	1	
Vertical corneal diameter, mm			
Mean	10.3±0.7	10.5±0.9	
Median (Q1, Q3)	10.5 (10.0, 10.5)	10.5 (10.0, 11.0)	0.245
Missing or unmeasurable	3	3	

CF: counting fingers. *Mann–Whitney *U* test

Table S3. Visual Outcomes for Persistent Fetal Vasculature of Previous Studies.

Study (year)	No. of eyes undergoing surgery and location	Median age at surgery (month)	Bilaterality	No. of eyes with final VA >20/400	No. of eyes with final VA > form vision*	Mean follow up duration (month)
Sisk et al. (2010)	26 anterior, 7 posterior, 37 combined	2.75	3 (4.3%)	15 (21.4%)	49 (70.0%)	47
Liu et al. (2017)	53 anterior, 16 posterior, 49 combined	22	13 (12.4%)	29 (24.6%)	Not reported	17
Yeh et al. (2019)	3 posterior, 22 combined	15	8 (44.4%)	2 (8.0%)	17 (68.0%)	41
Khandwala et al. (2021)	4 anterior, 1 posterior, 41 combined	10.1	1 (2.2%)	8 (17.4%)	24 (52.2%)	33
Current study	11posterior, 40 combined	6	7 (15.9%)	7 (13.7%)	19 (37.3%)	68

VA: visual acuity

* Form vision: VA of counting fingers, ‘central, steady, and maintained’, ‘fix-and-follow’, or better
